# Diversity and abundance of birds in dumpsites of Afar region, Ethiopia: implication for conservation

**DOI:** 10.1186/s40850-023-00177-6

**Published:** 2023-08-31

**Authors:** Weldemariam Tesfahunegny, Alembrhan Assefa

**Affiliations:** 1 Ethiopian Biodiversity Institute, Mekelle Biodiversity Center, Animals Biodiversity Case Team, Mekelle, Tigray, Ethiopia; 2 Department of Biology, College of Natural and Computational Science, Aksum University, P.O. Box, 1010, Axum, Tigray, Ethiopia

**Keywords:** Afar region, Bird conservation, Diversity, Dumpsite, Threatened species, Urbanbird

## Abstract

**Background:**

Dumpsites play key role in conservation of birds. A study was conducted to assess the diversity and abundance of birds in dumpsites of Afar region, Ethiopia from August, 2019 to March, 2020, covering both the wet and dry seasons. A total of nine dumpsites that contain domestic wastes found in different towns of Afar region were selected. Point count technique was employed to identify and count the birds. Shannon-Weiner diversity index was used to estimate species diversity and Two-way ANOVA was used to test birds’ species richness and abundance variation across dumpsites and seasons.

**Results:**

A total of 48 bird species consisting of one endemic and 10 globally threatened species were recorded. Red-billed Quelea, Marabou Stork and Egyptian Vulture were the most abundant species. There was a significant difference in bird species richness and abundance among dumpsites (F = 8.44, df = 8, P < 0.05) and F = 15.507, df = 8, P < 0.05), respectively. Moreover, a significant difference in abundance was also observed between the two seasons (F = 19.339, df = 1, P < 0.05). The highest species diversity (H’ = 3.18) was observed during wet season in Abala and Afambo dumpsites and the highest species evenness (E = 0.86) was observed during dry season in Afdera.

**Conclusion:**

Bird species diversity and abundance among dumpsites was high, and man-made disturbance are main threats for conservation of birds in the area. Therefore, conservation measures are needed to maintain survival of birds mainly the globally threatened species. Moreover, proper management of dumpsites is vital to support the waste dependent birds.

## Background

In biodiversity conservation and management, it is important to have detailed information or data in the relationship between organisms and their surrounding environment [1, 2]. Today there is an increase the proportion of the earth’s surface converting to human-dominated urban areas, and this becomes an opportune to know how the wildlife communities adapt to the complex urban ecosystems [3, 4]. The ecological impact or effect of urbanization should have great emphasis especially with its fast conversion of the formerly wildlife habitats all over the world. Urban development has intense effects on the survival, distribution, and abundance of wildlife and their habitats (5–7). Urbanization is mostly distinguished by fast population growth and high land use change, which are the main cause of biodiversity loss [5, 6].

Land use changes due to human-induced activities have radically changed the world’s biodiversity, and have been implicated as a major cause of declines in wildlife species [3]. As a result of natural habitat loss or disturbance, high numbers of wildlife species are found outside of the protected areas in artificial habitats such as farmlands and urban areas, and these artificial areas are found useful for wildlife with sufficient food sources [4]. Although urbanization is considered as the main threat to biodiversity, urban areas play a significant role in the conservation of diverse wildlife species including birds [3, 4, 7–9]. Of all wildlife, birds are the most common animals that can survive in highly sophisticated urban environments [7–12]. Urban environments provide birds with considerable amounts of food and water sources, roosting and nesting sites [12–15]. Birds have adapted to life in urban areas and search for food and shelter in different urban landscapes such as dumpsites, slaughterhouses, gardens, urban parks, open markets and restaurants [12, 16, 17].

Dumpsites are considered as a main topographical feature of urban areas, and they provide a wide variety of habitats that deliver roosting, nesting sites and other facilities, which are important for the survival of birds [15, 18]. Dumpsites contain various food items for birds such as organic scrap foods, bones, offal, insects, small mammals, dead animals, animal wastes and others [7, 19] and are considered to be on the factor attracting the generalist and scavenging bird species [18, 20]. They can be key feeding habitats of birds when properly managed and human activities have a great influence on attracting bird species through an accumulation of waste products like solid waste. These sites are found in the municipal areas and are used for disposal of the unwanted and used products that are generated by people living in urban areas. Birds regularly visit the dumpsites to eat and rest. However, little consideration has been given by urban planners to the sustainable implications in designing or planning for dumpsites in the conservation of urban birds. Globally, several studies found dumpsites provide positive impacts on birds creating suitable habitats as feeding sources [7, 9, 12, 18, 21].

In Ethiopia, several studies have been conducted on the diversity, distribution and abundance of bird fauna in different ecosystems particularly emphasize in protected areas including church forests [22–26]. However, the role of urban dumpsites for the conservation of bird communities is still not investigated as expected and only few studies have been done in major cities of the country [7, 12, 17, 20]. Study on bird diversity is valuable to identify the available species diversity and species that may be at risk and need more concerns, identify threats and prioritize the area for high conservation intervention strategies. The Afar region is an important staging point on the migration route to and from the Arabian Peninsula, and thus is used by many Palearctic species in spring and autumn [27]. The region hosts various protected areas to support diverse wildlife species however now a day due to different anthropogenic factors such as urbanization, industrialization, agricultural expansion, irrigation, road/highway construction and others the natural habitats are highly disturbing and deteriorating. As a result, some of the wildlife such as birds forced to move into other alternative and artificial habitats such as in urban environments. To the best of our knowledge, there was no study conducted on diversity of birds in dumpsites of Afar region, and therefore, this study aimed to assess the diversity and abundance of bird fauna in the unprotected dumpsites of the Afar region, Ethiopia.

## Materials and methods

### Description of the study area

**Fig. 1:**
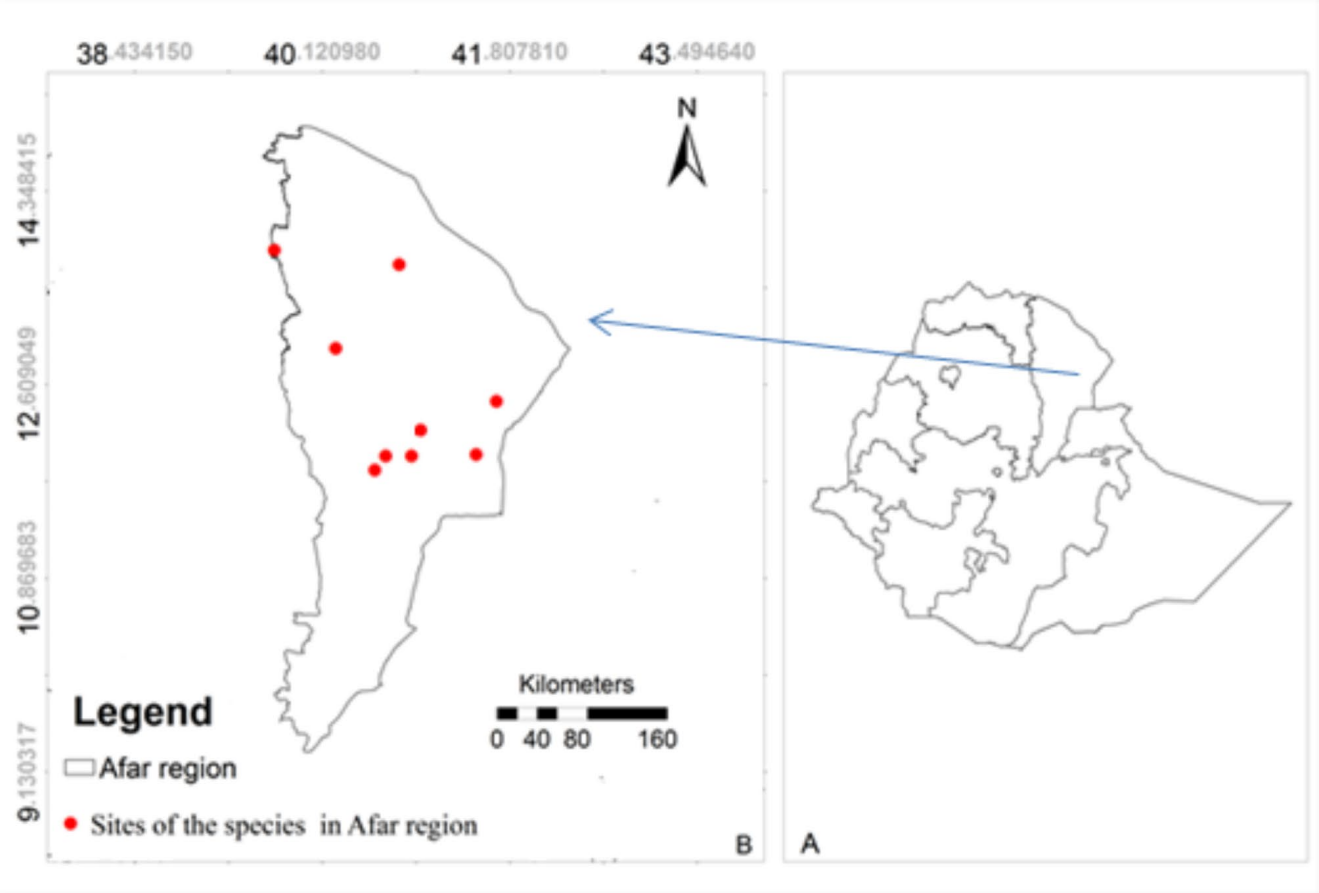
Map of (**B**) Afar region with study dumpsites and (**A**) Ethiopia with regions

The study was carried out in Afar region, northeastern Ethiopia. Afar region is geographically located at 9° 49’ 29.64” to 14° 30’ 21.38” N latitude and 39° 74’31.28” to 42° 28’ 13.14” E longitude (Fig. 1). This region is found in eastern lowlands of Ethiopia, and holds the world deepest part i.e. the Afar depression (125 meter below sea level). Afar region is bordered with Djibouti in east and Eritrea in north. It also bordered with Ethiopia’s Somalia, Oromia, Amhara and Tigray regions. Most parts of the region are below 1,000 meter above sea level (m.a.s.l). Afar region is largely desert scrubland with shallow salty lakes and long chains of volcanoes. It is the hottest part of Ethiopia with a mean annual temperature of 31^0^ C and the mean maximum temperature arrives up to 41^0^ C in June, and the mean minimum temperature ranges from 21 to 22 ^0^ C between November and December [28]. Its rainfall is sparse and irregular, and ranges from 200 to 560 mm per year [29]. The region is a semi-arid dryland and its vegetation cover consists of patches of scattered dry shrubs, acacia woodlands, bushland, grassland and wooded grassland [30].

The livelihood of the Afar people is mostly dominated by pastoralism, and pastoralists in the region raise cattle, goats and sheep but the agro-pastoralism is being promoted by the government. Settlement of the people of Afar in one particular area has been started lately and the urbanization has expanded rapidly since the 1980s [31]. Now day, they are increasingly moving and living in towns instead of continuing to embrace their traditional nomadic lifestyle. Afar region holds large livestock populations characterized by high annual death rate due to diseases and other reasons (> 418, 828 animals) and slaughter (101, 754 animals), which serve as source food for scavenging bird species [32]. The region is an important corridor for trans-boundary migratory birds to and from the Arabian Peninsula and hosts the highest number bird communities mainly the scavenging bird species such as the Egyptian Vultures [33–35]. Moreover, the unique topographic features and vegetation cover contribute to have many bird species in the region [34]. The region holds four recognized Important Bird Areas (IBA) of the country such as Lake Abe wetland complex, Aliyu Amba-Dulecha, Awash River valley and Yangudi-Rassa National Park, and other areas that meet the criteria for IBA. It also hosts different protected areas include Yangudi-Rassa National park, Awash National Park, Awash West, Alledeghi wildlife reserve, Gewani and Miele-Sardo wildlife sanctuaries and reserves, Afdem-Gewani Controlled hunting area and several forests to support substantial number of wildlife species [33, 36]. However, due to various anthropogenic activities such as agricultural expansion, industrialization and urbanization habitats loss is severely occur [37] and this situation affects wildlife such as birds and leads their movement towards unprotected artificial areas like dumpsites, which needs scientific investigations to perform conservation intervention strategies in these new artificial areas.

### Methods of data collection

A preliminary survey was conducted in July 2019 to gather basic information about the study dumpsites of the region such as the topographic features, plant coverage, animals like bird species, climatic conditions and locations, as well as to decide on the survey design and sampling strategy. A total of nine dumpsites were selected for the study that are found in different towns of the Afar region particularly at Logiya, Semera, Teru, Dufti, Abala, Afambo, Miele, Asaita and Afdera towns. These dumpsites hold domestic wastes that can attract high bird species due to the presence of sufficient food sources. So they were purposely selected based on their heterogeneity and integration as main components of feeding and roosting sites of birds. Pre-determined transect route was conducted throughout the dumpsites. A point count method was used to collect data on birds in the dumpsites. During the point count method, suitable locations or observation points were selected and birds were identified and counted from a fixed position within a 30 m radius for a specific period of 15 min at every point [38]. All birds observed within this 30 m radius were recorded. A total of three to five point count stations were established in each dumpsite depending on their size and maximum distance where observation of birds was possible. To minimize disturbance during counts, a waiting period of 3 to 5 min prior to counting was applied [22]. Field data collection was carried out from August to October, 2019 (wet season) and from January to March, 2020 (dry season). Data were collected every two weeks twice a day early in the morning from 6:00 a.m. to 10:00 a.m. and late afternoon from 4:00 p.m. to 6:00 p.m. when bird activity was high [39]. Binoculars and naked eyes were used for bird observation, and then bird species were identified and taxonomically classified using standard field guides [40, 41]. Photographs were also taken to confirm the identification of some of the species.

### Data analysis

Data were analyzed using SPSS version 20 software. Species diversity indices analysis and species accumulative curve were made using PAST software version 2.17 [42]. Relative abundance of bird species was determined using encounter rates that give basic ordinal scales of abundance (abundant, common, frequent, uncommon and rare) [43]. Encounter rate for each species was calculated by dividing the number of birds recorded by the number of hours spent searching, giving a figure of birds per hour for each species. The abundance categories were: < 0.1, 0.1-2.0, 2.1–10.0, 10.1–40.0 and > 40. For each category, the following abundance score is given: 1 (rare), 2 (uncommon), 3 (frequent), 4 (common) and 5 (abundant), respectively. Moreover, to assess bird community similarity among dumpsites, Simpson’s Similarity Index was applied, and calculated as: SI = 2 C/A + B. Where, SI = Simpson’s Similarity Index, C is the number of species the two communities have in common, A is the total number of species found in community A, and B is the total number of species found in community B [44]. Two-way ANOVA was used to test the significant variation of bird species richness and abundance across dumpsites and seasons.

## Results

### Species composition

**Fig. 2:**
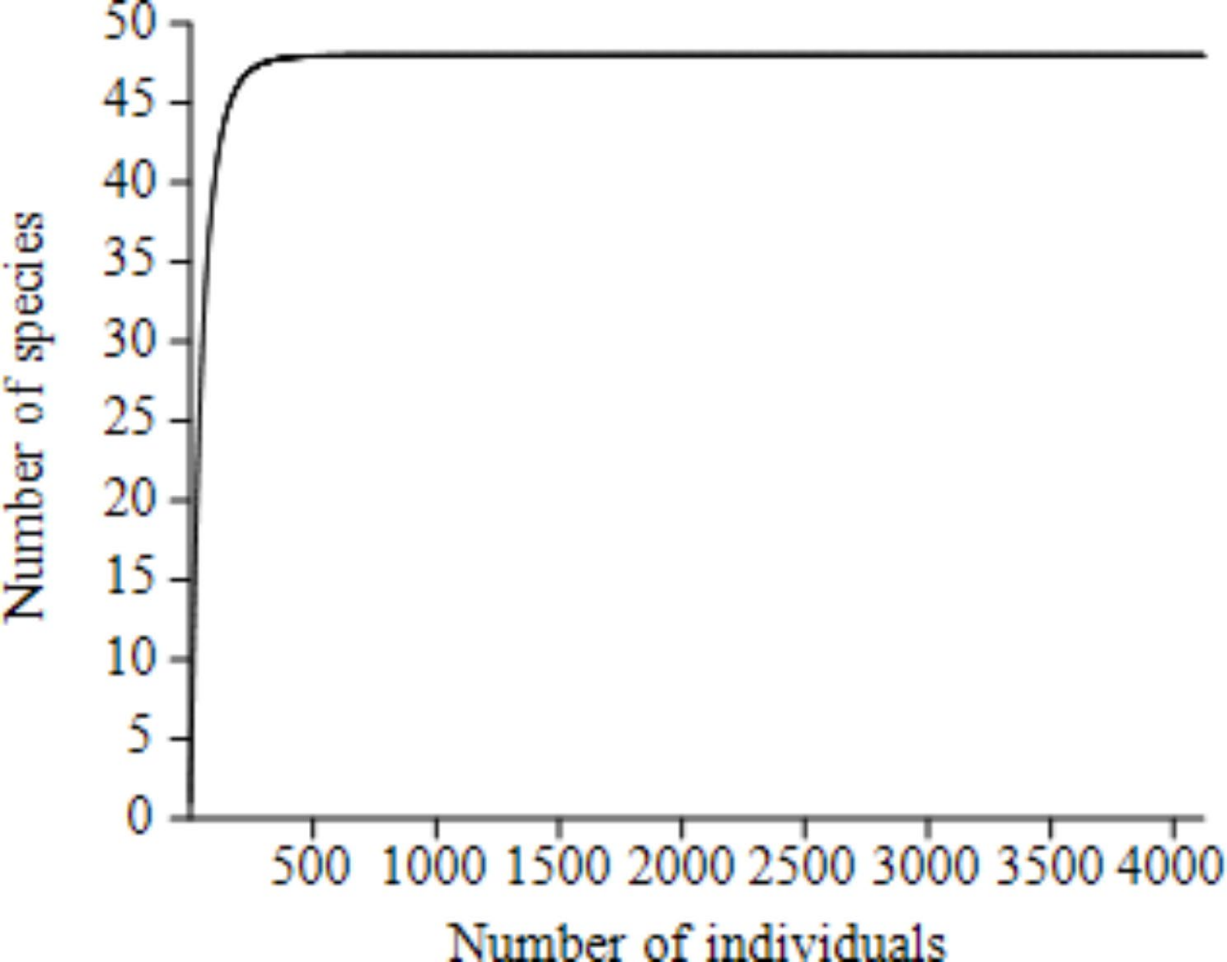
Species accumulative curve of birds in dumpsites of Afar, Ethiopia

**Fig. 3:**
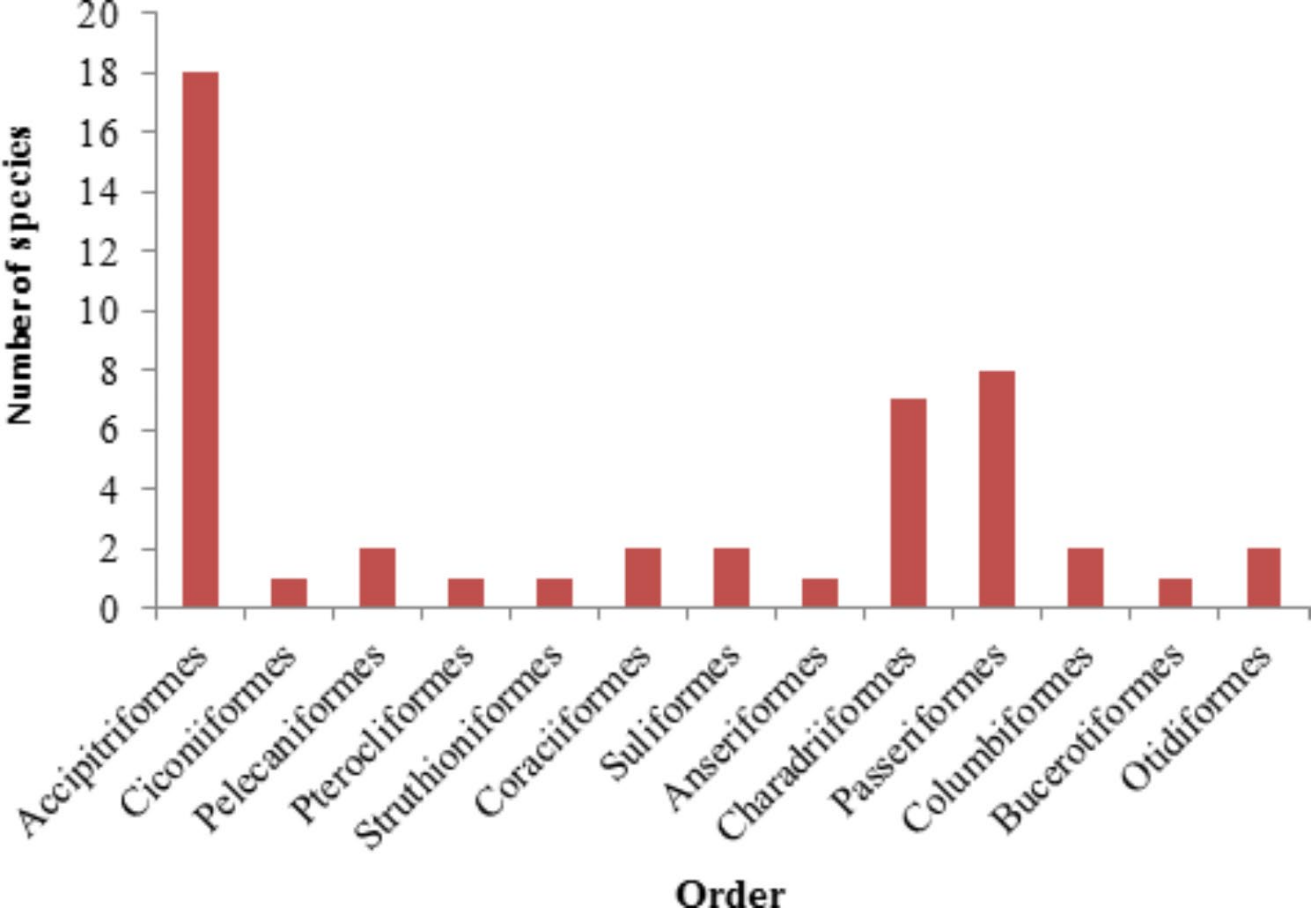
Number of bird species within their respective order in dumpsites of Afar, Ethiopia

A total of 48 species of birds belonging to 25 families and 13 orders were recorded during the study period (Table 1). The species accumulative curve of the dumpsites (Fig. 2) has fully reach asymptote showing that completeness of the survey and no more sampling effort is needed to explore the expected bird species of the study area. Among the 13 identified orders, Accipitriformes (37.5%) was the dominant order, containing a high number of species followed by the order Passeriformes (16.7%) and Charadriiformes (14.6%), while the remaining orders were represented by two and one species (Fig. 3). Accipitridae was the most dominant family (15 species) followed by Charadriidae (four species) and Corvidae (three species). Four families were represented by two species, and the remaining 18 families were represented only by a single species. Of the total identified 48 species, one endemic species to Ethiopia and Eritrea (Thick-billed Raven), four critically endangered species (Hooded Vulture, Rüppell’s Griffon, White-headed Vulture and White-backed Vulture), two endangered species (Egyptian Vulture and Lappet-faced Vulture), one vulnerable species (White-tailed Swallow) and three near-threatened species (Lammergeier, Kori Bustard and Arabian Bustard) were recorded in the study area. Among the 48 bird species recorded, 41 species were recorded during the wet season and 37 species were during the dry season. Among them, 30 species were common to both seasons, while 11 species were recorded only during the wet season and 7 species were also recorded only during the dry season. In addition, other wildlife species including Warthog (*Phacochoerus africanus)*, Crested Porcupine *(Hystrix cristata*), Common Jackal (*Canis aureus*), Gazelle Dorcas (*Gazella dorcas*), Sacred Baboon (*Papio hamadryas*), Guereza (*Colobus guereza*), Ground Squirrel (*Xerus rutilus*), feral cat (*Felis catus*) and domestic dog (*Canis lupus familiaris*) were also observed in the dumpsites.

**Table 1:** Bird species recorded in dumpsites of Afar, Ethiopia

Order	Family	Common name	Scientific name	Status	RA
Accipitriformes	Accipitridae	Egyptian Vulture	*Neophron percnopterus*	EN	3.81
		Griffon Vulture ^D^	*Gyps fulvus*	LC	2.11
		Lappet-faced Vulture ^D^	*Torgos tracheliotos*	EN	1.92
		Hooded Vulture	*Necrosyrtes monachus*	CR	2.93
		Rüppell’s Vulture ^W^	*Gyps rueppellii*	CR	3.03
		White-headed Vulture ^D^	*Trigonoceps occipitalis*	CR	2.62
		White-backed Vulture	*Gyps africanus*	CR	3.3
		Lammergeier ^W^	*Gypaetus barbatus*	NT	2.16
		Black Kite	*Milvus migrans*	LC	2.01
		Yellow-billed Kite	*Milvus aegyptius*	LC	2.55
		Montagu’s Harrier	*Circus pygargus*	LC	1.67
		Common Buzzard	*Buteo buteo*	LC	1.55
		Tawny Eagle	*Aquila rapax*	LC	1.43
		African Hawk-Eagle	*Hieraaetus spilogaster*	LC	1.77
		African Fish Eagle ^D^	*Haliaeetus vocife*	LC	1.94
	Corvidae	Thick-billed Raven ^+^	*Corvus crassirostris*	LC	1.5
		Fan-tailed Raven	*Corvus rhipidurus*	LC	2.5
		Pied Crow	*Corvus albus*	LC	3.35
Ciconiiformes	Ciconiidae	Marabou Stork ^D^	*Leptoptilos crumenifer*	LC	3.83
Pelecaniformes	Threskiornithidae	African Sacred Ibis ^W^	*Threskiornis aethiopicus*	LC	3.37
	Ardeidae	Little Egret	*Egretta garzetta*	LC	2.57
Pterocliformes	Pteroclidae	Lichtenstein’s Sand Grouse	*Pterocles lichtensteinii*	LC	1.8
Struthioniformes	Struthionidae	Common Ostrich ^D^	*Struthio camelus*	LC	0.82
Coraciiformes	Coraciidae	Abyssinian Roller	*Coracias abyssinicus*	LC	1.99
	Meropidae	Northern Carmine Bee-eater ^W^	*Merops nubicoides*	LC	2.26
Suliformes	Phalacrocoracidae	Long- tailed Cormorant	*Microcarbo africanus*	LC	1.65
	Anhingidae	African Darter	*Anhinga rufa*	LC	1.92
Anseriformes	Anatidae	Egyptian Goose ^W^	*Alopochen aegyptiaca*	LC	3.35
Charadriiformes	Charadriidae	Super Winged Plover	*anellus spinosus*	LC	1.41
		Black-headed Lapwing	*Vanellus tectus*	LC	2.28
		Black-winged Lapwing	*Vanellus melanopterus*	LC	1.43
		Grey Heron ^W^	*Ardea cinerea*	LC	2.45
	Burhinidae	Spotted Thick-knee	*Burhinus capensis*	LC	1.14
	Scolopacidae	Common Sandpiper	*Actitis hypoleucos*	LC	1.11
	Recurvirostridae	Black-winged Stilt ^W^	*Himantopus himantopus*	LC	1.19
Passeriformes	Pycnonotidae	Dodson’s Bulbul	*Pycnonotus dodsoni*	LC	1.36
		Common Bulbul ^W^	*Pycnonotus barbatus*	LC	2.04
	Ploceidae	Northern Masked Weaver ^W^	*Ploceus taeniopterus*	LC	2.76
		Red-billed Quelea	*Quelea Quelea*	LC	4.58
	Hirundinidae	White-tailed Swallow ^W^	*Hirundo megaensis*	VU	1.65
	Motacillidae	Golden Pipit	*Tmetothylacus tenellus*	LC	1.67
	Sturnidae	Shelly Starling ^W^	*Lamprotornis shelleyi*	LC	1.58
	Estrildidae	Red-billed Fire Finch	*Lagonosticta senegala*	LC	1.46
Columbiformes	Columbidae	Ring-necked Dove	*Streptopelia capicola*	LC	1.53
		Namaqua Dove	*Oena capensis*	LC	1.79
Bucerotiformes	Bucerotidae	Von Der Decken’s Horn bill	*Tockus deckeni*	LC	1.21
Otidiformes	Otididae	Kori Bustard	*Ardeotis kori*	NT	0.85
		Arabian Bustard ^D^	*Ardeotis arabs*	NT	0.8

Where, W = species recorded only during wet season, D = species recorded only during dry season, + = endemic species, CR = critically endangered, EN = endangered, VU = vulnerable, LC = least concern, NT = near-threatened, RA = relative abundance, Unmarked species = recorded in both wet and dry seasons

### Distribution and abundance

**Fig. 4:**
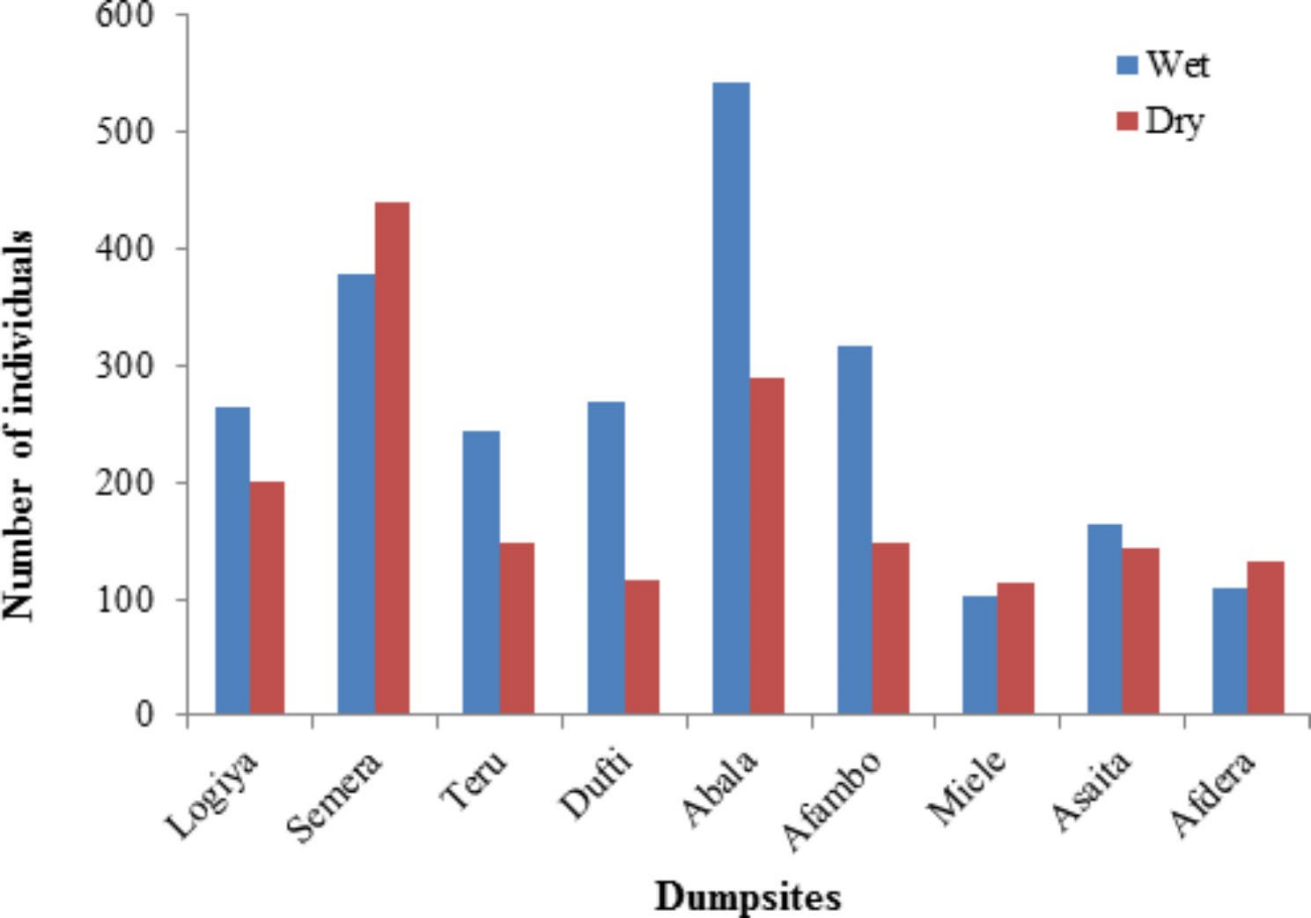
Abundance of birds in dumpsites of Afar during wet and dry seasons, Ethiopia

In this study, bird species distribution was varied among dumpsites. Among the total 48 species recorded, 42 species were found in Afambo dumpsite, 36 species in Teru, Dufti and Abala, 32 in Logiya and Asaita, 26 in Semera and Afdera, 20 in Miele dumpsite, and 20 species in all dumpsites. During this study, a total 4,123 individual of birds were counted. Of which, 2,392 individuals were recorded during the wet season and 1,731 were during the dry season, and this designated that relatively higher abundance of birds were recorded during the wet season. During wet season, Abala dumpsite (n = 543) had the highest abundance of birds followed by Semera (n = 379) and Afambo dumpsites (n = 317), respectively, while the least abundance was recorded from Miele dumpsite (n = 103). During the dry season, the highest number of individuals was recorded from Semera dumpsite (n = 440), followed by Abala (n = 289) and Logiya (n = 201) and the least was from Miele (n = 113) (Fig. 4). There was a significant difference in bird abundance between wet and dry seasons (F = 19.339, df = 1, P < 0.05) and among dumpsites (F = 15.507, df = 8, P < 0.05). Red-billed Quelea (4.58%) was the most abundant species, followed by Marabou Stork (3.83%), Egyptian Vulture (3.81%), African Sacred Ibis (3.37%), Pied Crow and Egyptian Goose (n = 3.35% for each), respectively, while Common Ostrich (0.82%) and Arabian Bustard (0.8%) were among the least abundant species (Table 1).

### Relative abundance

The relative abundance scores of bird species among dumpsites during the wet and dry seasons were described in Table 2. Most of the identified bird species were uncommon species followed by frequent and common species. During the wet season, the highest number of uncommon species was recorded in Asaita dumpsite (n = 18), whereas the least was in Semera (n = 4), for frequent species the highest number was in Abala (n = 18) and the least was in Afdera and Miele dumpsites (n = 4 for each), and for common species the highest number was found in Semera and Abala dumpsites (n = 3). During the dry season, the highest number of uncommon species was recorded in Afambo (n = 25) and the least was in Semera (n = 4), for frequent species the highest was in Semera (n = 15) and the least was in Dufti dumpsite (n = 3). During this study rare and abundant bird species were not registered during both seasons.

**Table 2:** Relative abundance of birds in dumpsites of Afar during wet and dry seasons, Ethiopia

Dumpsites	Season	Rank		
		Uncommon	Frequent	Common
Logiya	Wet	14	9	1
	Dry	17	8	1
Semera	Wet	4	13	3
	Dry	4	15	2
Teru	Wet	16	10	1
	Dry	21	5	-
Dufti	Wet	16	11	1
	Dry	22	3	-
Abala	Wet	11	18	3
	Dry	16	10	1
Afambo	Wet	15	17	-
	Dry	25	7	-
Miele	Wet	13	4	-
	Dry	8	7	-
Asaita	Wet	18	6	-
	Dry	22	4	-
Afdera	Wet	17	4	-
	Dry	11	9	-

#### Species diversity indices

Variations in bird species richness and species diversity were observed among dumpsites between wet and dry seasons (Table 3). During the wet season, Abala and Afambo dumpsites had the highest species richness (S = 32 each), followed by Dufti and Teru with 28 and 27 species, respectively. During dry season, the highest species richness was recorded from Afambo dumpsite (S = 32), followed by Abala (S = 27) and Logiya (S = 26). Miele dumpsite had the least species richness in both wet season (S = 17) and dry season (S = 15). There was a significant difference in bird species richness among dumpsites (F = 8.44, df = 8, P < 0.05). However, the species richness of birds did not show a significant difference between the two seasons (P > 0.05). In most (6) dumpsites species diversity was higher during the wet season compared to the dry season. During the wet season, the highest bird species diversity (H’ = 3.18) was recorded in both Abala and Afambo dumpsites followed by Teru (H’ = 2.91) and the least was in Miele (H′ = 2.59). During the dry season, the highest value of species diversity was recorded in Afambo dumpsite (H′ = 3.13) followed by Abala (H’ = 2.96) and the least was recorded in Miele (H’ = 2.51). During the wet season, the highest species evenness was recorded in Afdera (E = 0.84) and the lowest was in Dufti (E = 0.63). During the dry season, the highest and lowest species evenness were recorded in Afdera (E = 0.86) and Logiya (E = 0.66), respectively.

**Table 3:** Diversity indices of birds in dumpsites of Afar during wet and dry seasons

Dumpsites	Season	S	N	D	H’	E
Logiya	Wet	24	264	0.91	2.77	0.66
	Dry	26	201	0.912	2.84	0.66
Semera	Wet	20	379	0.929	2.81	0.83
	Dry	21	440	0.921	2.76	0.76
Teru	Wet	27	243	0.927	2.91	0.68
	Dry	26	147	0.932	2.95	0.74
Dufti	Wet	28	269	0.917	2.87	0.63
	Dry	25	117	0.928	2.93	0.77
Abala	Wet	32	543	0.948	3.18	0.75
	Dry	27	289	0.932	2.96	0.72
Afambo	Wet	32	317	0.949	3.18	0.75
	Dry	32	148	0.941	3.13	0.71
Miele	Wet	17	103	0.905	2.59	0.78
	Dry	15	113	0.907	2.51	0.82
Asaita	Wet	24	164	0.913	2.78	0.67
	Dry	26	143	0.909	2.87	0.68
Afdera	Wet	21	110	0.933	2.87	0.84
	Dry	20	133	0.936	2.85	0.86

Where, S = species richness, N = abundance, D = Simpson’s dominance index, H’= Shannon-Weiner diversity index and E = Evenness.

### Species similarity indices

Bird species similarity was varied across dumpsites between the two seasons (Table 4). During the wet season the highest species similarity (SI = 0.93) was recorded between Dufti and Abala dumpsites, while the least (SI = 0.39) was recorded between Teru and Asaita dumpsites. During the dry season the highest species similarity was recorded in between Teru and Abala dumpsites (SI = 0.98) and the least was between Logiya and Teru, and Teru and Asaita with a value of 0.58 each (Table 5). The overall similarity of species across all dumpsites is very low (SI = 0.15), which is < 25%, and this indicated that there is very less similarity of bird species among dumpsites.

**Table 4:** Species similarity of birds among dumpsites of Afar during wet season, Ethiopia

Dumpsites	Logiya	Semera	Teru	Bufti	Abala	Afambo	Miele	Asaita	Afdera
Logiya	-	0.82	0.43	0.46	0.54	0.54	0.73	0.92	0.84
Semera	-	-	0.51	0.54	0.62	0.46	0.86	0.82	0.73
Teru	-	-	-	0.91	0.92	0.85	0.55	0.39	0.5
Dufti	-	-	-	-	0.93	0.83	0.58	0.42	0.49
Abala	-	-	-	-	-	0.84	0.69	0.54	0.60
Afambo	-	-	-	-	-	-	0.49	0.54	0.64
Miele	-	-	-	-	-	-	-	0.73	0.84
Asaita	-	-	-	-	-	-	-	-	0.84
Afdera	-	-	-	-	-	-	-	-	-

**Table 5:** Species similarity of birds among dumpsites of Afar during dry season, Ethiopia

Dumpsites	Logiya	Semera	Teru	Bufti	Abala	Afambo	Miele	Asaita	Afdera
Logiya	-	0.89	0.58	0.59	0.60	0.72	0.73	0.88	0.87
Semera	-	-	0.64	0.65	0.67	0.60	0.83	0.89	0.73
Teru	-	-	-	0.94	0.98	0.89	0.73	0.58	0.61
Dufti	-	-	-	-	0.96	0.88	0.7	0.59	0.67
Abala	-	-	-	-	-	0.92	0.71	0.6	0.64
Afambo	-	-	-	-	-	-	0.64	0.72	0.69
Miele	-	-	-	-	-	-	-	0.73	0.8
Asaita	-	-	-	-	-	-	-	-	0.87
Afdera	-	-	-	-	-	-	-	-	-

## Discussion

This study presents the first survey of birds in dumpsites of the region, and a total of 48 species of birds were identified. This indicates dumpsites provide sufficient feeding, roosting and nesting sites for a large number of bird species, and this implies that dumpsites play a key role in the conservation of bird fauna. Dumpsites of the Afar region are characterized by having domestic wastes such as food waste, paper, metals, plastics, glass and animal wastes including bone, offal and even dead cattle. Due to the high death rate of livestock population mass of carcass bodies of dead animals is available on dumpsites, which lead to attract substantial number of bird species mainly the scavenging species. The wastes in dumpsites cause to present different animals like insects and small mammals such as rodents, which are potential food for birds. Moreover, the presences of some trees in and around dumpsites and nearby buildings also contribute for roosting of birds. In recent times, high habitat loss has been recorded in the region that caused by various man-made and natural factors including industrialization, urbanization, agricultural expansion, drought, flood, desert locusts, severe wind event, and introduction of invasive alien plant species like the Prosopis, which causes significant impacts on biodiversity and ecosystem services [37, 45]. As the declines or disturbances of the natural habitats such as natural forests, national parks, wildlife sanctuaries and reserves for birds, they have become increasingly dependent on artificial habitats such as dumpsites and these sites are found for birds with high food sources and acts as their alternative feeding, roosting and nesting sites. This result in line with the findings of previous studies notified the availability of organic food sources in dumpsites is one of the most important factors influencing the survival and distribution of birds in urban areas [17–19]. Aschalew et al. [46] and Zerihun et al. [47] also revealed that both feeding and nesting sites are the main factors in determining the species diversity of birds. Similarly, studies from Africa, Asia and America also declared that dumpsites are alternative promising sites for high bird diversity [9, 18, 19, 21].

However, this result was in contrast with the reports of Abeba et al. [11] and Matejczyk et al. [48] who reported that due to the existence of different detrimental non-biodegradable materials such as metals, plastics, glasses, paints, electric wires, various toxics and hazardous pathogens in dumpsites make to harbor low species diversity and abundance of birds. Likewise, a recent study indicates that plastic wastes and debris have great effect on the health of aquatic and terrestrial bird species [49]. The macro-plastic and micro-plastic wastes, and their derived additives and absorbed chemicals have harmful effects on the survival, feeding, growth, development, reproductive output and physiology of birds since they accumulate in their body parts. Birds are well known to ingest waste pollution, especially plastic. Plastic can block the gastrointestinal tract, leading to chocking. Ingested plastic can make birds feel full, reducing nutrition and caused starvation [50]. As plastic breakdown, trace elements and toxins can be released, damaging vital organs. Although the study dumpsites have different non-degradable materials, the bird species diversity is high and this might be indicates the edible waste materials that can attract birds are highly available but detail studies are demanding to evaluate plastic pollution burden and toxicological/health effects of chronic plastic exposure of birds especially the globally threatened species for the better conservation effort.

The number of species recorded during this study was relatively consistent with the report of Yrgalem et al. [12] who recorded 30 bird species from dumpsites of Wolkite town, Southern Ethiopia. However, it was lower and higher as compared to studies conducted in Bahir Dar city (186 species) [11] and in Dire Dawa city (6 species) [17], respectively. Bird species richness variation among different localities might be due to the variation in quantity and quality of food sources, human disturbances, climatic conditions and other environmental factors such as altitude, vegetation cover and water availability. This study has recorded 10 globally threatened scavenging bird species. This might associated with presence sufficient food sources in the area. The Afar region is distinguished by having a large population of cattle and other animals like goat and sheep however unlike other regions of the country there is high death rates (11%) of livestock population per year [33]. This might be contributing the high availability of food for scavenging birds. Kendall et al. [51] stated that the quantity of prey mortality is an important driver of vultures habitat use than prey abundance. Arkumarev et al. [34] reported that the Afar region in Ethiopia hosts high congregation of Egyptian vultures in Eastern Africa. However, birds of the region are exposed to various threats include poisoning, electrocution, use of pesticides, habitat loss and overgrazing [34, 45]. Studies in Afar indicate that there is frequent dead of birds specially raptor species on dumpsites [34, 35]. The most serious potential threats for the raptors in the region seem to be non-intentional poisoning. Poison baits with strychnine are widely used by veterinarians in the region targeting stray dogs; this common practice poses a high risk of poisoning and secondary poisoning for the scavenging birds. The poisoned stray dogs dumped unburied at the dumpsites and this may cause mass poisoning of birds such as vultures as they tend to congregate around carcasses bodies of dead animals. Another potential threat to the raptors of the region is electrocution [34]. Birds mostly vultures are susceptible to electrocution due to their habit of roosting and perching on powerline pylons. Hence, the deteriorating conservation status of the scavenging birds seems mainly connected to incidental mortality from feeding on poisonous baits or deliberately poisoned carcasses to control animals considered as pests or harmful to the society. Moreover, there is high land degradation/ habitat loss and overgrazing practices in the region could also consider as threats to the bird communities.

Across dumpsites, the highest and lowest number of species was recorded in Afambo and Miele dumpsites, respectively, and this is maybe due to the availability of high food resources, less human disturbance and existence of trees around that offer forage and nest for species in Afambo dumpsite, whereas Miele dumpsite is highly disturbed area since crossed by the Ethiopia-Djibouti highway and also has less vegetation cover as compared to the other dumpsites. The highest abundance of birds was recorded in Abala dumpsite and followed by Semera and the least abundance was in Miele dumpsite. The variation in species richness and abundance among dumpsites may be due to the variation of food sources, roosting and nesting sites, vegetation cover and human disturbance. Semera and Abala dumpsites are broader than other dumpsites, and this could be contribute to exist more scrap foods, dead animals, and other resources such as trees, poles and buildings around for their feeding, roosting and nesting purpose since they are found in highly populated large towns. Blackwell et al. [52] described that bird species composition and abundance is highly affected by the availability of resources which is essential for species, and mostly birds need undisturbed areas for feeding, resting and nesting. Human pressures in and around dumpsites have profound effect on the abundance of birds and as human disturbance increases, birds move away from the areas they exist [7, 17, 53, 54]. Similarly, other authors also notified that ecological factors such as availability of food, human disturbance and other climatic conditions can make variations in bird species abundance across sites [55, 56].

Higher number of bird species richness and abundance were observed during wet season than dry season, which in line with the reports of many authors who reported that bird species richness and abundance was higher during the wet season than during the dry season in different parts of Ethiopia [20, 57–59]. However, the present study contradicts the findings of Yrgalem et al. [12] and Amare and Girma [60] that birds species richness and abundance was the highest during the dry season. The maximum number of bird species and abundance during the wet season compared to the dry season may be due to the presence of high food sources, favorable weather conditions and occasionally high quality of nesting and breeding sites. Ramprakash and Purohit [61] conveyed that vultures abundance display high levels of seasonal variations to meet their requirement for nest, roost and food. Various researches indicated that seasonal changes leads variation in the availability of water and food resources, and consequently, birds change across habitats depending on their needs and accessibility of cover and food [16, 20, 62].

From the identified bird species, Red-billed Quelea was the most abundant species in the study area followed by Marabou Stork and Egyptian Vulture, respectively. Red-billed Quelea is mostly recognized as the most populous species on earth, and high abundance of this species in the area might be probably related with the social and gregarious habit of the species during flying, nesting and feeding. Most of the scavenging bird species include vultures, eagles, kites, crows, ravens, buzzards and others were more abundant and found in all dumpsites of the region, and this might be related with their strong feeding potentials since they feed on other animal dead remains, generalist feeding habit and high adaptations against human interferences. This shows that dumpsites serve as bird reservoirs however since they consist of various domestic harmful wastes they may have a negative impact on the survival and conservation of the highly threatened bird species and this requests to have proper management of dumpsites. The high relative abundance of Egyptian Goose and African Sacred Ibis in some dumpsites of the study area might be associated with the presence of lakes, wetlands and other aquatic ecosystems along dumpsites that can create food sources for these non-scavenger birds.

Most of the bird species in this study were found within the ordinal rank of uncommon. This might be related to the wide habitat range and more niche specialty of the species. Similarly, some authors have reported the occurrence of more uncommon bird species in different localities of Ethiopia [7, 58, 63]. Ryan and Owino [64] stated that the wide habitat coverage, niche requirement, breeding nature of the species and destruction of habitat might be a reason for the species to be found as uncommon in a particular area. Rare and abundant bird species were not recorded in both wet and dry seasons of this study, and this is consistent with the report of Tamenut and Fasika [26] from Shoa, Ethiopia.

The highest species diversity indices were recorded in Afambo and Abala dumpsites, and the highest species evenness was found in Afdera. The variation in species diversity and evenness at different dumpsites might be due to the difference in availability of food sources, roosting and nesting sites as well as level of human disturbances. A study by Megersa et al. [63] and Haider et al. [65] indicated that the difference in species diversity and evenness of birds are highly related with variations in habitat features, feeding behaviors and level habitat disturbance. In this study, the highest species similarity was observed between Abala and Dufti dumpsites during the wet season and between Abala and Teru dumpsites during the dry season, and this might be related to these dumpsites are found in nearby geographical locations with similar ecological features and equivalent level of human interferences. The least bird species similarity between various dumpsites might be due to habitat-specific variations in feeding adaptation and the response of birds to various man-made disturbances. This result agrees with the findings of Tamenut and Fasika [26], Tamenut et al. [58] and Megersa et al. [63] who reported that animals in the same ecological condition are highly similar to each other in terms of species richness and topographical structure than animals in different environmental conditions.

## Conclusion

The present study indicates dumpsites of the Afar region are identified for their high bird species (48) with a relatively high abundance for each species. This implies other ecologically and economically important fauna may be using the region, and this also confirms that the dumpsites of the region are critical environments for wildlife conservation. The distribution of diverse bird species among dumpsites is attributable to the presence of sufficient feeding, nesting and roosting sites. More importantly, the death of a high livestock population enables the region to harbor relatively high bird species diversity. However, various anthropogenic activities include human disturbance, free ranging domestic dogs and cats, disposing of non-edible waste products like glasses, paints, metals, toxics and hazardous pathogens, poisoning of dead animals, electrocution and highway vehicles collision are negatively affecting the diversity and abundance of birds. Therefore, conservation measures are required to protect the bird diversity. In addition, proper management and protection of dumpsites is highly recommended to support the waste dependent birds as well as further investigations are critical specifically for the globally threatened species from chemical poisoning in the freely discarded wastes.

## Data Availability

All the data generated and analyzed during this manuscript preparation are available on the hands of the corresponding author.

## Abbreviations

ANOVA: Analysis of variance
PAST: Paleontological Statistics
SPSS: Statistical Product Services and Solutions
